# Comparison of the Potential Relative Bioaccessibility of Zinc Supplements—In Vitro Studies

**DOI:** 10.3390/nu15122813

**Published:** 2023-06-20

**Authors:** Justyna Ośko, Wiktoria Pierlejewska, Małgorzata Grembecka

**Affiliations:** Department of Bromatology, Medical University of Gdańsk, Al. Gen. J. Hallera 107, 80-416 Gdańsk, Poland; wiktoria.pierlejewska@gumed.edu.pl

**Keywords:** bioaccessibility, zinc, dietary supplement, in vitro, FAAS

## Abstract

The aim of this study was to determine the potential relative bioaccessibility of zinc (Zn) from selected dietary supplements during in vitro digestion. The bioaccessibility of Zn was evaluated in dietary supplements differing in the pharmaceutical form, content, dose, and chemical form of the element. The content of Zn was determined by flame atomic absorption spectrometry. The applied method was validated, and results were characterised by good linearity (R^2^ = 0.998), recovery (109%), and accuracy (0.02%). As a result of the tests conducted, it was found that the bioaccessibility of Zn from dietary supplements varied and ranged from 1.1% to 9.4%. The highest bioaccessibility was found for zinc diglycinate and the lowest for zinc sulphate. In 9 out of 10 tested dietary supplements, the determined Zn content was higher than the one declared by the producer (up to 161%). The estimated tolerable upper intake level (UL) was exceeded by five of the analysed dietary supplements (123–146%). The analysed dietary supplements were assessed in terms of compliance with the information contained on the product packaging, based on current Polish and European legal regulations. The qualitative assessment was performed according to the United States Pharmacopoeia (USP) guidelines.

## 1. Introduction

**Figure 1:**
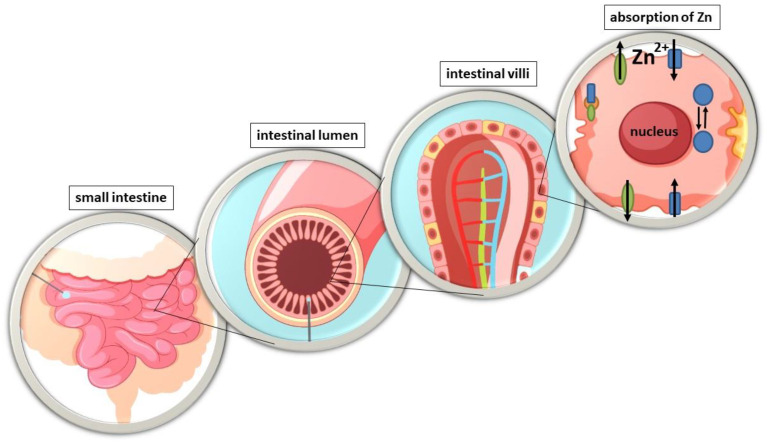
Zinc absorption in the intestinal tract [5].

Zinc (Zn) is one of the most important micronutrients, which plays a key role in many principal biological processes in the human body [1,2]. To compensate for the loss of Zn from the body and to ensure its homeostasis, a constant supply from food is essential [3]. Zinc is absorbed throughout the small intestine [4], but the main site of its intestinal absorption in humans is still debated [5]. In rat studies, the highest degree of absorption has been reported in the duodenum and ileum [6] or only in the ileum [7] or jejunum [4], respectively. There are only a few in vivo studies showing the actual site of Zn absorption in humans. However, through the use of small intestinal perfusion techniques in healthy individuals, it has been found that the main sites of absorption in the human intestines are both the duodenum [8] and the jejunum [4]. Zinc uptake takes place at the intestinal brushtail membrane, where it is transported from the lumen to the lymphatic epithelial cells, the enterocytes (Figure 1). Subsequent excretion of the cation on the basolateral side of the enterocytes releases it into the portal blood, where it is mainly bound to albumin, which distributes the metal in the body [9]. An unbalanced status or deficiency of this microelement is associated with serious health consequences, resulting in high morbidity [10].

According to the World Health Organisation (WHO), one third of the world’s population is at risk of Zn deficiency, with serious health consequences such as reduced growth rate, impairments of immune defence, impaired taste, wound healing, diarrhoea, or alopecia [11]. Zinc is one of the key cofactors of important enzymes that contribute to the proper functioning of the antioxidant defence system. This element contributes to the protection of cells against free radical damage. By providing membrane stabilisation, it inhibits the enzyme nicotinamide adenine dinucleotide oxidase (NADPH-Oxidase), a pro-oxidant enzyme, and induces the synthesis of metallothionein [12]. Metallothionein is responsible for participating in the reduction of free radicals and the binding of reactive oxygen species that are formed in stressful situations [13,14]. Long-term Zn deficiency contributes to increased vulnerability to damage mediated by oxidative stress. There is an increased level of lipid peroxidation in mitochondrial and microsomal membranes, as well as osmotic fragility of erythrocyte membranes [14]. The metallothionein present is responsible for the maintenance and control of Zn concentration in every cell of the body. Both Zn deficiency and Zn excess are pro-oxidant states [15]. Excess Zn is mainly associated with impaired copper homeostasis [10].

The absorption of dietary Zn in humans is usually in the range of 16–50% [16], which is inversely proportional to oral Zn intake [17]. Furthermore, net absorption is regulated by its homeostasis in the body and, therefore, depends on individual Zn status, which adjusts to a long-term Zn-poor diet. Absorption of this element also depends on its concentration and increases with increased dietary intake up to a maximum value [18]. In addition, its level can affect its absorption. Individuals with Zn deficiency absorb it with increased efficiency, whereas those on a Zn-rich diet show reduced absorption efficiency [9]. Phytates, cellulose, iron, cadmium, and tin have a limiting effect on the absorption of this metal from the diet. Of particular importance in the diet is phytate, which forms stable complexes with Zn in the intestinal lumen, making it an insoluble and biologically inaccessible compound [17]. Therefore, an estimate of the phytate/zinc molar ratio was made, by which the quality of a mixed diet as a source of Zn can be estimated [19]. A ratio with a value of <5 indicates a good dietary source of Zn, while a value in the range 5–15 indicates moderate bioavailability. In contrast, a phytate/zinc ratio >15 indicates poor bioavailability, and consumption of this diet may result in a negative balance of the element [20]. The estimated physiological requirement for bioavailable zinc at 1 g phytate becomes twice as high as for a diet without phytate [21]. Fractional Zn absorption also depends primarily on its intake, as its efficacy decreases with increasing intake [16,22]. In addition, the different chemical forms of this metal affect its intestinal absorption, which is of particular importance for Zn supplements [23].

In most cases, Zn supplementation combined with dietary sources is a convenient option to compensate for insufficient intake, malabsorption, or increased loss due to intestinal diseases [24]. This metal absorption depends not only on adequate dietary intake, but also on its intestinal availability with food. Further investigation of the influence of these factors on Zn absorption by the intestinal epithelium remains one of the present research topics [16,25].

More and more attention has been given to developing suitable in vitro models to mimic in vivo processes. In recent years, in vitro screening methods have been developed and refined to determine the bioavailability of nutrients from food. There are currently four methods to measure/estimate bioaccessibility and/or bioavailability in vitro: solubility, dialysability, or a gastrointestinal model (e.g., TIM) for bioaccessibility and Caco-2 models for bioavailability [26]. These methods can provide a wealth of useful information, especially when the multitude of factors influencing nutrient absorption are taken into account. Bioavailability is defined as the amount of an ingested nutrient that is absorbed and available for physiological functions that depend on digestion, release from the food matrix, absorption by intestinal cells, and transport to body cells. Bioaccessibility, on the other hand, is the amount of a nutrient that is potentially available for absorption and is dependent solely on digestion and release from the food matrix [27]. In vitro bioavailability/bioaccessibility testing methods are extremely useful for assessing nutrient interactions, the influence of luminal factors, food preparation and processing practises, and food matrix characteristics [27]. Due to the high cost of research, the maintenance of appropriate ethical standards in animal studies, and the benefits of in vitro models providing a microenvironment that accelerates the study of cellular processes at the molecular level [28,29], the latter has become an increasingly popular model for bioaccessibility studies. Although in vitro digestion models have their disadvantages, e.g., they cannot mimic the complex dynamics of the digestive process or the physiological interaction with the host, they also have great advantages over more complex methods. They are characterised by simplicity, low cost, good extrinsic and inter-laboratory reproducibility, and easy evaluation of the different phases of digestion, making these models suitable for mechanistic studies, hypothesis building, and screening [30]. In vitro methods are usually the methods of choice because they avoid the need to submit animal protocols, eliminate or reduce the need for laboratory personnel experienced in working with animals, and are economical [31].

Due to the significant consumption of dietary supplements, the aim of the study was to evaluate the bioaccessibility of Zn from selected dietary supplements available on the Polish market. The influence of the chemical form of the compound found in the supplement on bioaccessibility was also assessed. Moreover, the percentage of the tolerable upper intake level of Zn, established by Scientific Opinion of Dietary Reference Values by EFSA (10 October 2014) [32], was estimated. The analysed dietary supplements were assessed in terms of compliance with the information contained on the product packaging, based on current Polish and European legal regulations [33,34,35]. Due to the lack of European guidelines for the assessment of the variability in the weight, shape, and size of the form of dietary supplements, a qualitative assessment was performed according to the guidelines of the United States Pharmacopoeia (USP) [36], the Food and Drug Administration (FDA) [37], and Overgaard et al. [38].

## 2. Materials and Methods

### 2.1. Samples, Reagents, and Standards

The analysed dietary supplements samples (tablets and capsules) were purchased from physical and internet pharmacies in Poland. All dietary supplements currently available on the Polish market containing only Zn in their composition were selected for the study. In total, 10 dietary supplements from different producers were tested, i.e., 30 analytical samples were prepared and analysed for Zn content. The products differed in their pharmaceutical form, composition, content of active ingredients, and chemical form of Zn (Table 1). Samples (capsules and tablets) were stored in string bags at room temperature (25 °C). Certified reference material of Polish Virginia Tobacco Leaves (INCT-OBTL-6) was used for accuracy and precision evaluation. Samples were analysed in triplicate.

### 2.2. Determination of Zn Content Using the FAAS Method

The concentration of Zn was determined by atomic absorption spectrometry with an air–acetylene flame (FAAS) using the deuterium background correction (λ = 213.9 nm). The analysis was performed using Thermo Scientifics i3000 (Waltham, MA, USA). MiliQ water (18.2 MΩ/cm, Millipore, MA, USA) was used to prepare Zn standard (0.5 g/0.5 L, Merck^®^, Darmstadt, Germany) solution and reagents. Nitric acid 69% (Tracepure, Merck^®^, Darmstadt, Germany) and hydrochloric acid 36% (Tracepure, Merck^®^, Darmstadt, Germany) were used in mineralization process (described in Section 2.6).

### 2.3. Bioaccessibility of Zn

For bioaccessibility assays, gastrointestinal digestion solutions were prepared immediately before the analysis. The gastric solution was prepared by dissolving porcine pepsin gastric mucosa (Sigma-Aldrich^®^, St. Louis, MO, USA) in 0.1 mol/L HCl. The mixture of porcine pancreatin (Sigma-Aldrich^®^, St. Louis, MO, USA) and bile salts (Sigma-Aldrich^®^, St. Louis, MO, USA) was dissolved in 1 mol/L sodium bicarbonate (POCH, Gliwice, Poland) to form a mixture of intestinal solution. The PIPES buffer (Sigma-Aldrich^®^, St. Louis, MO, USA) was prepared by dissolving it in water, and the pH was adjusted to 7.5 with 3 mol/L HCl [39].

### 2.4. Preparation of Dialysis Membranes

Dialysis membranes (10 kDa, Sigma-Aldrich^®^, USA) were cut into 10 cm pieces and placed in a glass beaker. Then, they were washed twice with MiliQ water for 15 min. The beaker with membranes was placed in a water bath at 80 °C for 30 min to heat up. Then, the membranes were washed three times with MiliQ water. The membranes prepared in this manner were stored in MiliQ water until they were used for research [39].

### 2.5. In Vitro Gastrointestinal Digestion Simulation

**Figure 2:**
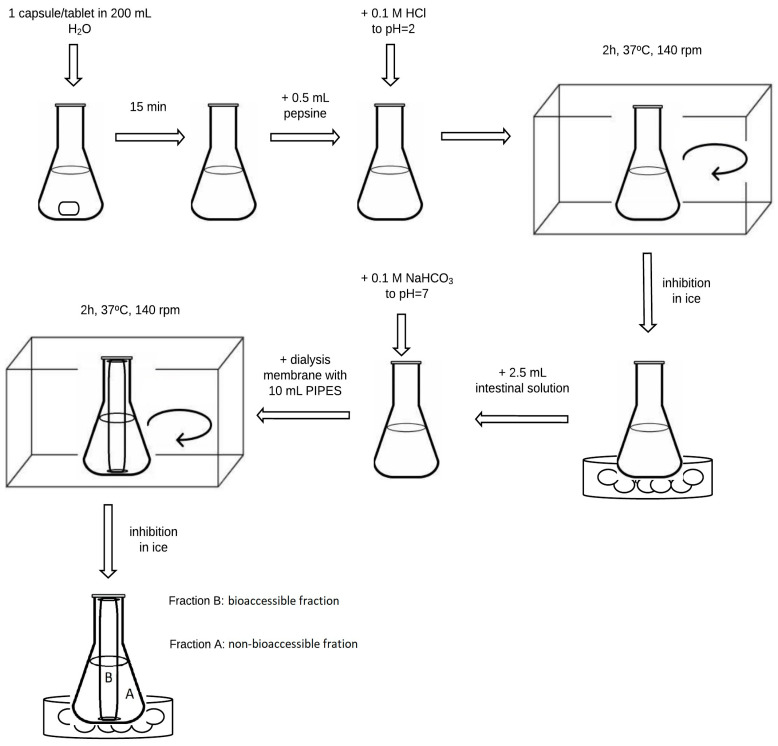
Scheme of the gastrointestinal digestion simulation method.

The in vitro gastrointestinal digestion simulation method was based on previous studies with our modifications [39,40,41], and is presented in Figure 2. One capsule of dietary supplement was weighed on an analytical balance (in triplicate) and transferred to a 250 mL conical flask. An amount of 200 mL of MilliQ water was dispensed into each flask and allowed to stand for 15 min. Then, 0.5 mL of the previously prepared pepsin solution was added to the flasks, and the pH was adjusted to 2 with 0.1 M hydrochloric acid solution. Samples were incubated in a water bath with a thermostated shaker at 37 °C for 2 h. After the incubation time, the samples were transferred to ice to inhibit the enzymatic reaction. In the next stage, 2.5 mL of intestinal juice was added. The contents of the flasks were adjusted to pH = 7 using a 0.1 M sodium bicarbonate solution. Then, 10 mL of PIPES buffer was pipetted into previously prepared membranes. The membranes’ endings were closed with dedicated clips. The membranes were placed in flasks and again incubated in a shaking water bath for 2 h at 37 °C. Subsequently, the samples were placed on ice to inactivate the enzymes. Each sample was transferred to quartz crucibles and evaporated to dryness in a boiling water bath. A blind test was performed for each series, proceeding as if with a dietary supplement sample.

### 2.6. Mineralization

The mineralization process was carried out by incinerating the dry residue in quartz crucibles in a muffle furnace using a gradient temperature increase to 540 °C. The resulting ash was treated with 1.5 mL of a 36% HCl solution and 3 drops of a 63% HNO3 solution. The treated ash was then evaporated on a boiling water bath. Next, 1.5 mL of 36% HCl solution was added to the samples, which were covered with a watch glass and heated for about 1 min. The crucible content was quantitatively transferred to 25 mL volumetric flasks and replenished with MiliQ water [42]. All the samples were prepared accordingly to be analysed by FAAS as specified in Section 2.2.

### 2.7. Method Validation

The limit of detection (LOD) and limit of quantification (LOQ) for Zn were calculated based on the independently prepared blank samples’ measurements. According to the method described by Konieczka and Namieśnik [43], LOD was set to blank means + 3SD, where blank mean is a result of all blank samples’ measurements and SD is their standard deviation, whereas LOQ was calculated by multiplying LOD by a factor of three. The LOD and LOQ for fraction A (subject to passing through the membrane) were 0.05 µg/mL and 0.16 µg/mL, respectively, while for fraction B (imitating the gastrointestinal lumen) (Figure 1), the LOD was 0.04 µg/mL and the LOQ 0.13 µg/mL.

The reliability of the method was determined using the certified reference material, i.e., Polish Virginia Tobacco Leaves (INCT-PVTL-6), and the accuracy was 109% and the precision 0.02%.

### 2.8. Evaluation of Dietary Supplements according to Polish and European Regulations and the Requirements of the United States Pharmacopoeia (USP 43-NF 38)

The dietary supplements analysed were assessed for correct labelling in accordance with Polish and European regulations. The manufacturer’s declaration on the packaging was verified based on the Act of 25 August 2006 on food and nutrition safety (Art. 48) [34], the Regulation of the Minister of Health of 9 October 2007 on the composition and labelling of dietary supplements [35], and the Regulation of the European Parliament and of the Council (EU) No 1169/2011 of 25 October 2011 on the provision of food information to consumers, including Articles 17, 18, 21–27, and 29 [33]. The dietary supplements were assessed in terms of form (<2091> chapter “Weight variation of dietary supplements”) in light of the United States Pharmacopoeia (USP 43-NF 38) [36] and shape and size according to the guidelines of the Food and Drug Administration (FDA) [37] and Overgaard et al. [38]. Measurements of size were taken using a digital calliper (measuring range: 0–150 mm, resolution: 0.01 mm, Parkside, OWIM GmbH & Co. KG, Neckarsulm, Germany) model: HG00962A (0.01 mm). The weight of the dietary supplements tested was measured on an analytical balance with a resolution of ±0.0001 g (semi-micro balance TS2215Di, VWR, Leuven, Belgium).

## 3. Results and Discussion

### 3.1. Bioaccessibility of Zn and Influence of Its Form

The conducted tests allowed for the assessment of the in vitro bioaccessibility of Zn from dietary supplements. Among the most commonly used in vitro methods to assess zinc bioaccessibility are dialysability methods. Both solubility and dialysis estimation methods aim to estimate the bioaccessibility or fraction of the element available for absorption [26]. These methods provide preliminary information that can be used to conduct further in-depth in vivo studies. Chiplonkar et al. [27], for example, used published data on the absorption of Zn by humans from various meals. They then correlated the percentage susceptibility of Zn to in vitro dialysis with absorption in humans. The results showed that the in vitro dialysability method was consistent with the human absorption data with a coefficient of 0.925 (*p* < 0.001).

The analysed products differed in their pharmaceutical form, composition, Zn content, and chemical form (Table 1). The results of studies on the Zn content in the “A” fraction (imitating the gastrointestinal lumen) and the “B” fraction (subject to passage through the dialysis membrane) and the potential relative bioaccessibility of this element are presented in Table 2. The analysed supplements were characterised by varied chemical forms. Zinc content in the “A” fraction was in the range 10.2–35.9 mg/capsule and the lowest value was found for the DS2 preparation while the highest was for the DS5. The content of this element in the “B” fraction ranged from 0.19 mg/capsule (DS3) to 2.42 mg/capsule (DS1) (Table 2).

The potential relative bioaccessibility of Zn varied from 1.13 to 9.38%. The highest value was found for DS2-9.38%, and the lowest for DS3 (1.13%). Due to the digestion process, many different Zn forms are present in the intestine, complexed by food-derived macromolecules or low-weight molecular ligands [9]. Hence, the availability and accessibility of Zn depend on its solubility and the stability of the respective complexes in the intestinal lumen. This is influenced by diet as well as physiological factors such as mucus and intestinal fluid. Together, these factors alter the speciation of the ion as well as its free and available concentration in the lumen, which consequently affects its absorption by the intestinal epithelium [5]. For example, iron inhibits Zn when it is consumed in the form of dietary supplements in the absence of other foods and when the molar ratio of Zn is 25:1 [44]. Similarly, phytates and nucleic acids (containing phosphorus) reduce Zn absorption. Calcium, on the other hand, can potentially reduce absorption of this element, but only when phytate is present in the food [45].

The selected dietary supplements were characterised by different chemical forms of Zn (Tabel 1). The highest bioaccessibility was determined for DS2 (Table 2) which contained Zn in the form of diglycinate. However, given the low dose of zinc diglycinate in DS2, it can be assumed that a relatively high solubility after digestion occurs. Other products with the same chemical form also showed higher bioaccessibility (7.86% for DS1, 6.59% for DS10, and 5.77% for DS9) as compared to other chemical forms of the studied dietary supplements (Table 2). The relative bioaccessibility of Zn in the form of gluconate amounted to 4.48% and 6.19% in the DS7 and DS8, respectively. Lower bioaccessibility results were obtained for the dietary supplements DS3, DS4, DS5, and DS6, in which Zn was present in the form of zinc methionine, zinc picolinate, zinc citrate, and zinc sulphate (1.13%, 1.99%, 3.15%, and 3.38%, respectively). Wegmuller et al. [46] used ^67^Zn and ^70^Zn as dual isotopic tracer methods to examine the absorption of Zn from various forms. By comparing zinc citrate, gluconate, and zinc oxide, they found that zinc citrate was as well absorbed by healthy adults as zinc gluconate. The authors highlighted that the use of zinc citrate could provide an alternative to the more expensive form (zinc gluconate) or forms that give a strongly metallic, bitter, and astringent taste (zinc sulphate and acetate), which would need to be masked in a supplement. Guillem et al. [47], on the other hand, in a study of infant milk, found less susceptibility to in vitro dialysis of zinc citrate compared to zinc gluconate and zinc oxide.

Interestingly, the dietary supplement DS3 (15 mg Zn/capsule) had the lowest bioaccessibility compared to the supplement with the highest estimated relative bioaccessibility vs. a lower Zn content (10 mg/capsule). It was probably due to the chemical form, zinc sulphate, in which this element was bound in the supplement.

Organic zinc salts such as acetate and lactate, in particular amino acid chelates, have higher bioaccessibility than zinc sulphate [48]. Similar conclusions were presented in the work of Schlegel and Windisch [49], who studied the bioaccessibility of glycinate- and sulphate-bound forms of Zn in rats. They showed that the bioaccessibility of the zinc glycinate complex is even 30% higher than that of zinc sulphate. A similar study was conducted by Sapota et al. [50], who compared the absorption of two forms of Zn (zinc sulphate and zinc gluconate) in rats with prostate cancer. Based on the results, the authors concluded that zinc gluconate had higher bioavailability at low doses compared to zinc sulphate, suggesting the consideration of zinc gluconate supplementation in men. According to a published report by Brown et al. [23], it is recommended for the formulation of multicomponent supplements to provide readily bioavailable zinc salts in the form of sulphate (ZnSO_4_), zinc gluconate, or zinc acetate, as these are more efficient for absorption.

### 3.2. Realisation of Reference Intake of Zn

According to the Scientific Opinion on Dietary Reference Values for zinc (10 October 2014, EFSA), which is 25 mg/day for adults, a percentage of tolerable upper intake level (UL) of this element was calculated [32]. The estimated UL percentage showed that half of the dietary supplements analysed containing Zn (DS1, DS5, DS6, DS7, and DS9) exceeded the allowed level (123–146%) (Table 2). Zinc supplementation is recommended for Acrodermatitis enteropathica, Wilson’s disease, diarrhoea, and leprosy. For the occurrence of diarrhoea in children, the recommended daily dose can range from 10 mg/day (children <3 years) to 20 mg (children >3 years) [51]. According to studies [52,53], zinc supplementation is effective in reducing the severity and duration of acute and persistent diarrhoea. Also, supplementation appears to be valuable in preventing COVID-19-related deaths. In an ongoing meta-analysis evaluating the effect of zinc supplementation on SARS-CoV-2 mortality, it was found that zinc supplementation led to a significantly lower risk of death compared to the control group [54]. According to data published by Haase et al. [24] following a large number of studies on the efficacy of zinc supplementation, it can only be used in a limited number of diseases.

### 3.3. Assessment of the Manufacturer’s Declaration in Light of Current Legislation

**Figure 3:**
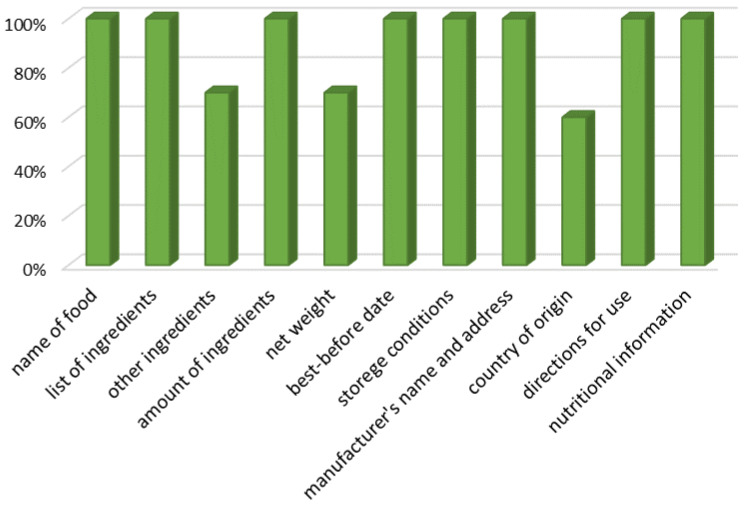
Graph for assessing the conformity of information on packaging.

In accordance with Polish regulations, the tested dietary supplements were verified for compliance with the information contained on the packaging. According to the information that is required on the packaging, such as the name of the food, list of ingredients, other ingredients (e.g., excipients, substances causing allergies, intolerances, etc.), the specified amount of ingredients, net weight, best before date, storage conditions, name and address of the manufacturer, country of origin, instructions for use, and nutritional information, among others, were verified in the dietary supplements tested. Three supplements out of ten did not have net weight information or information on other ingredients. Four of the products analysed also lacked information on the country of origin of the product (Figure 3). All dietary supplements analysed complied with the conditions of correct formulation and labelling: the term “dietary supplement”, the recommended portion to be consumed, information on the content of the ingredients in relation to the reference daily intake values (%), a warning not to exceed the recommended daily portion, a statement that the dietary supplement must not be used as a substitute for a varied diet, and appropriate storage conditions (including non-availability to young children).

Eight out of ten dietary supplements contained a health claim, while one contained a health and nutrition claim. More than half of the products (6) did not contain additives in the formulation, i.e., titanium dioxide. This substance in 2021, according to an opinion published by the European Food Safety Authority (EFSA), was identified as a compound of uncertain safety due to its possible genotoxic effects [55].

### 3.4. Evaluation of Dietary Supplements against the Requirements of the FDA and the United States Pharmacopoeia (USP 43-NF 38)

The size and shape of the dietary supplement form were evaluated using the criteria outlined by the FDA [37]. Twenty individual capsules and tablets were measured for each product. For capsules, length and width (=depth) were measured, and for round tablets, length (=width) and depth were measured. The results are shown in Table 3.

**Figure 4:**
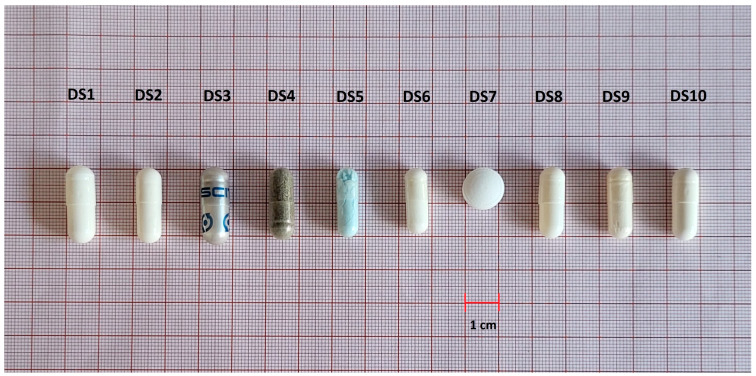
Variation of sizes and shapes of the analysed dietary supplements.

Of the dietary supplements analysed in terms of size of pharmaceutical forms, all met FDA recommendations. The predominant shape among capsules was the cylindrical form, and tablets had the round form (Figure 4).

The requirements of the US Pharmacopoeia in <2091> chapter “Weight variation of dietary supplements” were used to assess the weight variation of the analysed products. The weight was measured by weighing 20 individual capsules and tablets. The average weight for each product was calculated (Table 4). According to pharmacopoeial requirements, the highest and lowest capsule weight values must be within 90–110% of the average capsule weight. For tablets, the limit set was 5–10% for mass values, where 2 out of 20 tablets weighed could not exceed this limit. However, the size of both the tablet and capsule is important for oesophageal passage. Channer and Virjee [56] conducted a study on the passage time of 8 mm and 11 mm round tablets, showing that tablets of smaller size had a shorter passage time. This is of particular importance for people who have difficulty swallowing [57].

As a result of the assessment of weight uniformity according to pharmacopoeial requirements, the limit for DS7 supplement in tablet form was found not to be met. The differences in individual tablets compared to the average weight of the weighed 20 tablets were more than the designated limit (5–10%). Therefore, the supplement does not meet the weight uniformity requirement for the pharmaceutical form. In the case of the evaluation of the manufacturer’s declaration in terms of the weight of tablets/capsules in relation to the measured values, one dietary supplement out of three achieved a declaration percentage of 117%.

## 4. Conclusions

The potential relative bioaccessibility of Zn from dietary supplements and its content in the dialysis membrane (which was equivalent to the intestine) as well as outside it (a place imitating the lumen of the gastrointestinal tract) were highly varied. The highest bioaccessibility was determined for DS2 (9.38%) while the lowest was for DS3 (1.13%). Studies on the potential relative bioaccessibility of Zn from dietary supplements have shown variation in this parameter, which could be influenced by many factors, such as the chemical form of Zn complexes, which have different solubility and stability, and the amount of the substance in the dietary supplement. Zinc diglycinate proved to be the chemical form with the highest bioaccessibility, while zinc sulphate had the lowest. The bioaccessibility of Zn is not directly proportional to its dose in the dietary supplement. Exceeding Zn content in accordance with the manufacturer’s declaration in the dietary supplements analysed resulted in a high percentage of UL realisation, which amounted to 123–146% for half of the products analysed. Therefore, knowledge of Zn bioaccessibility from complex food matrices can be included in dietary surveys. Most of them currently only consider the total Zn content of an adequate diet. The evaluation of dietary supplements in terms of meeting the requirements of correct labelling, uniformity of weight of the pharmaceutical form, size, and shape is proving to be extremely important in assessing the quality of products that bear a significant resemblance to pharmaceutical drugs. The establishment of Polish regulations in this area would contribute to a better quality of the food products on offer and to minimising the risk of danger associated with their consumption.

## Figures and Tables

**Table 1:** Characteristics of dietary supplements.

	Pharmaceutical Form	Zn Content Declared by Producer	Chemical Form	Dosage
DS1	Capsules	25 mg	zinc bisglycinate	1 capsule/day
DS2	Capsules	10 mg	zinc bisglycinate	1 capsule/day
DS3	Capsules	30 mg	zinc sulfate	2 capsules/day
DS4	Capsules	15 mg	zinc picolinate	1 capsule/day
DS5	Capsules	25 mg	zinc citrate	1 capsule/day
DS6	Capsules	30 mg	zinc methionine	1 capsule/day
DS7	Tablets	24.6 mg	zinc gluconate	1 tablet/day
DS8	Capsules	15 mg	zinc gluconate	1 capsule/day
DS9	Capsules	30 mg	zinc bisglycinate	1 capsule/day
DS10	Capsules	15 mg	zinc bisglycinate	1 capsule/day

**Table 2:** Zinc content in dietary supplements [mg/capsule] and potential relative bioaccessibility [%].

Code	Fraction	Average Content [mg/Capsule] (Range)	Total Content ^a^ [mg]	Potential Relative Bioaccessibility [%]	Percentage of UL (for 1 Capsule/Tablet) ^b^ [%]
DS1	A	28.4 ± 4.90 (21.8–33.6)	30.8	7.86	123
	B	2.42 ± 0.008 (2.42–2.43)			
DS2	A	10.2 ± 1.11 (8.81–11.5)	11.3	9.38	45.2
	B	1.06 ± 0.24 (0.80–1.38)			
DS3	A	16.6 ± 1.64 (14.3–18.2)	16.8	1.13	67.2
	B	0.19 ± 0.12 (0.05–0.33)			
DS4	A	23.3 ± 2.29 (20.5–26.1)	24.1	3.15	96.4
	B	0.76 ± 0.05 (0.72–0.83)			
DS5	A	35.9 ± 10.8 (25.1–46.7)	36.6	1.99	146
	B	0.73 ± 0.01 (0.72–0.75)			
DS6	A	30.0 ± 0.67 (29.4–30.9)	31.1	3.38	124
	B	1.05 ± 0.003 (1.04–1.05)			
DS7	A	32.2 ± 4.03 (26.7–36.0)	33.7	4.48	135
	B	1.51 ± 0.002 (1.50–1.51)			
DS8	A	12.6 ± 1.09 (11.7–14.1)	13.4	6.19	53.6
	B	0.83 ± 0.13 (0.65–0.96)			
DS9	A	31.7 ± 0.77 (31.0–32.8)	33.6	5.77	134
	B	1.94 ± 0.10 (1.82–2.05)			
DS10	A	16.2 ± 0.71 (15.2–16.8)	17.3	6.59	69.2
	B	1.14 ± 0.13 (0.97–1.30)			

^a^ Sum of fraction A and B. ^b^ EFSA NDA Panel (EFSA Panel on Dietetic Products, Nutrition and Allergies), 2014. [32]; UL—tolerable upper intake level (25 mg/day for adults).

**Table 3:** Analysis of shape and size of the analysed dietary supplements.

Code	Length ± SD [mm]	Width ± SD [mm]	Depth ± SD [mm]	L + W + D ^1^	Shape	FDA Recommendation ^2^
DS1	23.1 ± 0.05	8.40 ± 0.08	8.40 ± 0.08	39.9	cylindrical capsule	acceptable
DS2	22.0 ± 0.36	7.53 ± 0.03	7.53 ± 0.03	37.1	cylindrical capsule	acceptable
DS3	23.3 ± 0.10	8.46 ± 0.06	8.46 ± 0.06	40.2	cylindrical capsule	acceptable
DS4	21.6 ± 0.17	7.63 ± 0.02	7.63 ± 0.02	36.9	cylindrical capsule	acceptable
DS5	20.51 ± 0.53	6.84 ± 0.05	6.84 ± 0.05	34.2	cylindrical capsule	acceptable
DS6	19.52 ± 0.32	6.90 ± 0.05	6.90 ± 0.05	33.3	cylindrical capsule	acceptable
DS7	12.3 ± 0.02	12.3 ± 0.02	6.83 ± 0.16	31.4	round tablet	acceptable
DS8	21.09 ± 0.10	7.57 ± 0.04	7.57 ± 0.04	36.2	cylindrical capsule	acceptable
DS9	21.13 ± 0.12	7.61 ± 0.03	7.61 ± 0.03	36.4	cylindrical capsule	acceptable
DS10	21.52 ± 0.05	7.56 ± 0.06	7.56 ± 0.06	36.6	cylindrical capsule	acceptable

^1^ Sum of length, width, and depth; ^2^ FDA recommends that “the largest tablet or capsule size should not exceed 22 mm, and capsules should not exceed 23.3 ± 0.3 mm in length and 8.56 mm in diameter”.

**Table 4:** Uniformity of weight according to pharmacopoeial requirements.

Code	Average Weight ± SD [mg]	Declared Weight [mg]	Percentage of Declaration [%]	Min ^1^–Max ^2^ [%]	Pharmacopeia Criteria
DS1	507 ± 6.97	500	101	98–102	passed
DS2	383 ± 8.59	NA	ND	95–105	passed
DS3	846 ± 44.2	NA	ND	94–110	passed
DS4	504 ± 13.3	NA	ND	95–105	passed
DS5	428 ± 13.7	NA	ND	94–105	passed
DS6	458 ± 10.0	NA	ND	96–105	passed
DS7	649 ± 48.8	NA	ND	87–120	failed
DS8	234 ± 9.70	200	117	93–109	passed
DS9	500 ± 8.36	ND	ND	96–104	passed
DS10	520 ± 3.97	490	106	99–101	passed

NA—not available, no information on the package; ND—no data, value cannot be calculated due to a lack of data from the manufacturer; ^1^ percentage specified for the lowest mass measurement values; ^2^ percentage specified for the highest mass measurement values.

## Data Availability

All data are contained within the article.
